# Social Ties and Depressive Symptoms Among Middle-Aged and Older Adults in China During Emergencies: The Moderating Role of Disaster Exposure

**DOI:** 10.3390/healthcare14172867

**Published:** 2026-09-06

**Authors:** Jingfang Liu, Peng Ding, Zijing Liu

**Affiliations:** 1 School of Management, Shanghai University, Shanghai 200444, China; 2 Antai College of Economics and Management, Shanghai Jiao Tong University, Shanghai 200030, China

**Keywords:** depressive symptoms, middle-aged and older adults, public health emergency, social ties, disaster exposure, life satisfaction

## Abstract

**Background:** Public health emergencies disrupt daily life and impose a heavy burden on mental health. Middle-aged and older adults are particularly vulnerable to the shocks caused by such emergencies, which may substantially increase their risk of depressive symptoms. Although social ties are closely related to mental health, little is known about how different types of social ties are associated with mental health in emergency contexts. Moreover, these associations have rarely been examined alongside broader contextual shocks. This study explores how different social ties are associated with lower depressive symptoms and examines the role of indirect disaster exposure. **Methods:** Data were drawn from the 2020 wave of the China Health and Retirement Longitudinal Study (CHARLS) and matched with urban cumulative confirmed cases. Using data from 15,897 respondents, we employed linear regression and bootstrap resampling to test whether life satisfaction statistically accounts for some of the associations between social ties, household financial stability, and depressive symptoms. The model also examined whether the association between life satisfaction and depressive symptoms varied with indirect disaster exposure. **Results:** Broader social participation (b = −0.242, *p* < 0.01), more positive perceived changes in intergenerational relationships (b = −0.536, *p* < 0.05), and better household financial stability (b = −1.637, *p* < 0.01) were associated with fewer depressive symptoms. Life satisfaction statistically contributed to the link between weak ties, financial instability, and depressive symptoms (b = −2.136, *p* < 0.01). Indirect disaster exposure strengthened the negative association between life satisfaction and depressive symptoms (b = −0.134, *p* < 0.01). **Conclusions:** During public health emergencies, depressive symptoms among middle-aged and older adults are associated not only with social ties and household financial stability, but also with the broader context of city-level disaster exposure. Depression prevention strategies need to consider both individual psychological resources and contextual disaster exposure to identify vulnerable populations and reduce mental health risk.

## 1. Introduction

### Public Health Emergencies, Social Ties and Depressive Symptoms

Globally, there were an estimated 1.17 billion cases of mental disorders across all ages and sexes in 2023, ranking the fifth leading cause of global disease burden. Major depressive disorder also showed a marked increase between 1990 and 2023 [1]. Public health emergencies such as the COVID-19 pandemic can disrupt daily routines, restrict social interaction, and create persistent uncertainty. These disruptions pose long-term risks for depression and other psychiatric conditions [2], particularly among middle-aged and older adults who are more likely to experience chronic illness, mobility limitations, financial strain, and reliance on family support [3]. Identifying factors associated with depressive symptoms in this population is therefore important for depression prevention during future emergencies.

Social ties may be an important factor. They are closely related to mental health in later life, but different types of social ties may operate through different pathways during emergencies. Strong ties such as intergenerational relationships demonstrate a significant negative correlation with depression risk among middle-aged and older adults [4]. Complementing strong ties, weak ties such as social activities are characterized by lower role obligations and provide a broader form of support that effectively alleviates depressive symptoms [5]. Loneliness and social isolation are distinct forms of social disconnection in later life, with both being associated with depression, anxiety, and cognitive impairment. In contrast, reliable social relationships provide emotional, informational, and practical support [6]. Financial resources may also affect depression by shaping perceived security and control, especially when public health emergencies disrupt household income and daily expenses [7].

Beyond the direct associations previously outlined, social ties and economic resources may also show indirect statistical associations with depressive symptoms involving life satisfaction. Grounded in Diener’s model of subjective well-being, life satisfaction is established as a fundamental component of psychological health [8]. Empirical evidence shows that both relational support and material resources are associated with higher life satisfaction [9]. Consequently, this elevated sense of satisfaction can function as a critical psychological asset which is linked to fewer depressive symptoms, a relationship further corroborated by research underscoring the buffering role of life satisfaction against psychopathology [10].

Disaster Exposure Matrix theory highlights the multi-dimensional nature of disaster exposure, distinguishing between direct and indirect forms of exposure [11]. However, existing research on disaster exposure has predominantly focused on the direct effects, such as the physical and immediate psychological impact on individuals [12]. These studies tend to treat disaster exposure as a direct predictor of mental health outcomes, overlooking the potential moderating role that indirect exposure plays in shaping the relationships between key predictors (e.g., social ties, financial stability) and depressive symptoms among middle-aged and older adults.

The Chinese context is particularly relevant to these relationships. The country is undergoing rapid population aging, making mental health in middle and later adulthood an increasingly important public health concern [13]. Within Chinese families, intergenerational relationships remain a central source of emotional, practical, and financial support, reflecting the continuing importance of filial responsibility in later life [4,14]. During the 2020 COVID-19 pandemic, lockdowns, mobility restrictions, and social-distancing measures restricted family contact and community participation. Longitudinal evidence from China indicates that older adults in regions with wider viral spread and stricter social-distancing measures experienced greater declines in life satisfaction [15]. Together with substantial variation in epidemic burden across cities, these circumstances provide an informative setting for examining how social ties and household financial circumstances were associated with depressive symptoms during the pandemic.

To address these gaps, this study integrates individual-level data from the 2020 wave of CHARLS, which is based on a nationally representative sampling design, with city-level disaster exposure statistics. It aims to investigate the patterns linking social ties (weak ties and intergenerational relationships) and household financial stability to depression among middle-aged and older adults during public health emergencies, particularly the COVID-19 pandemic. Specifically, the research examines the statistical indirect contribution of life satisfaction and further assesses whether the association between life satisfaction and depressive symptoms varies with indirect disaster exposure. The findings are expected to advance the social psychology of aging and inform targeted mental health policies for middle-aged and older adults in future large-scale crises.

## 2. Theoretical Basis and Hypothesis Proposal

### 2.1. Social Ties and Depression Among Middle-Aged and Older Adults During an Emergency

Social networks promote mental health by providing resources and buffering stress [16], functions that are carried by strong ties and weak ties to alleviate depression through different mechanisms. Strong ties are close relationships characterized by frequent interactions and emotional intimacy, such as those among family members and close friends. They are well-documented to protect mental health [17]. Weak ties typically involve less frequent contact, lower emotional intensity, and fewer role obligations, contributing to reducing depressive symptoms [5]. However, during emergencies, only limited work has modeled both strong and weak social ties. Hence, we propose the following hypotheses:

**H1a.**  *During an emergency, weak ties are negatively associated with depression levels among middle-aged and older adults.*

**H1b.**  *During an emergency, improvements in intergenerational relationships are negatively associated with depression levels among middle-aged and older adults.*

### 2.2. Household Financial Stability and Depression Among Middle-Aged and Older Adults in Emergencies

Household financial stability reflects whether household income is sufficient to cover daily expenses during an emergency. From the perspective of conservation of resources (COR) theory, individuals have a tendency to preserve and acquire resources, and the actual or potential loss of these resources poses a threat. Difficulty in meeting daily expenses represents a perceived threat to current resources and may lead to depression [18,19]. However, it has not yet been fully explored in the framework of public health emergencies. We therefore hypothesize the following:

**H1c.**  *During an emergency, household financial stability is negatively associated with depression levels among middle-aged and older adults.*

### 2.3. Cross-Sectional Indirect Associations Involving Life Satisfaction

Life satisfaction constitutes a fundamental component of subjective well-being [8]. It is well documented as a linking factor between adversity and psychological distress [20]. It reflects an individual’s overall cognitive evaluation of life circumstances and is closely related to mental health. Social ties and financial security are associated with life satisfaction through perceived support, control, and stability. Higher life satisfaction may in turn reduce vulnerability to depressive symptoms during public health emergencies. Therefore, this study examines whether life satisfaction statistically contributes to the associations between social and financial resources and depressive symptoms and hypothesizes:

**H2a.**  *Life satisfaction statistically accounts for part of the cross-sectional association between weak ties and depressive symptoms among middle-aged and older adults during an emergency.*

**H2b.**  *Life satisfaction statistically accounts for part of the cross-sectional association between changes in intergenerational relationships and depressive symptoms among middle-aged and older adults during an emergency.*

**H2c.**  *Life satisfaction statistically accounts for part of the cross-sectional association between household financial stability and depressive symptoms among middle-aged and older adults during an emergency.*

### 2.4. The Moderating Role of Indirect Disaster Exposure

In previous studies, disaster exposure has often been treated as a direct predictor of mental health outcomes. However, the indirect effects of disaster exposure, such as increased psychological stress due to social isolation, economic disruption, and uncertainty, have received limited attention. Building on the Disaster Exposure Matrix theory, this study treats indirect disaster exposure as a contextual condition that may shape the association between life satisfaction and depression. Middle-aged and older adults in cities with greater COVID-19 exposure may face stronger uncertainty, stricter public health measures and greater disruption to daily life. Under such conditions, life satisfaction may become more salient as a psychological resource. We therefore hypothesize:

**H3.**  *Indirect disaster exposure moderates the relationship between life satisfaction and depression, such that the negative association is strengthened in cities with higher indirect disaster exposure.*

**Figure 1:**
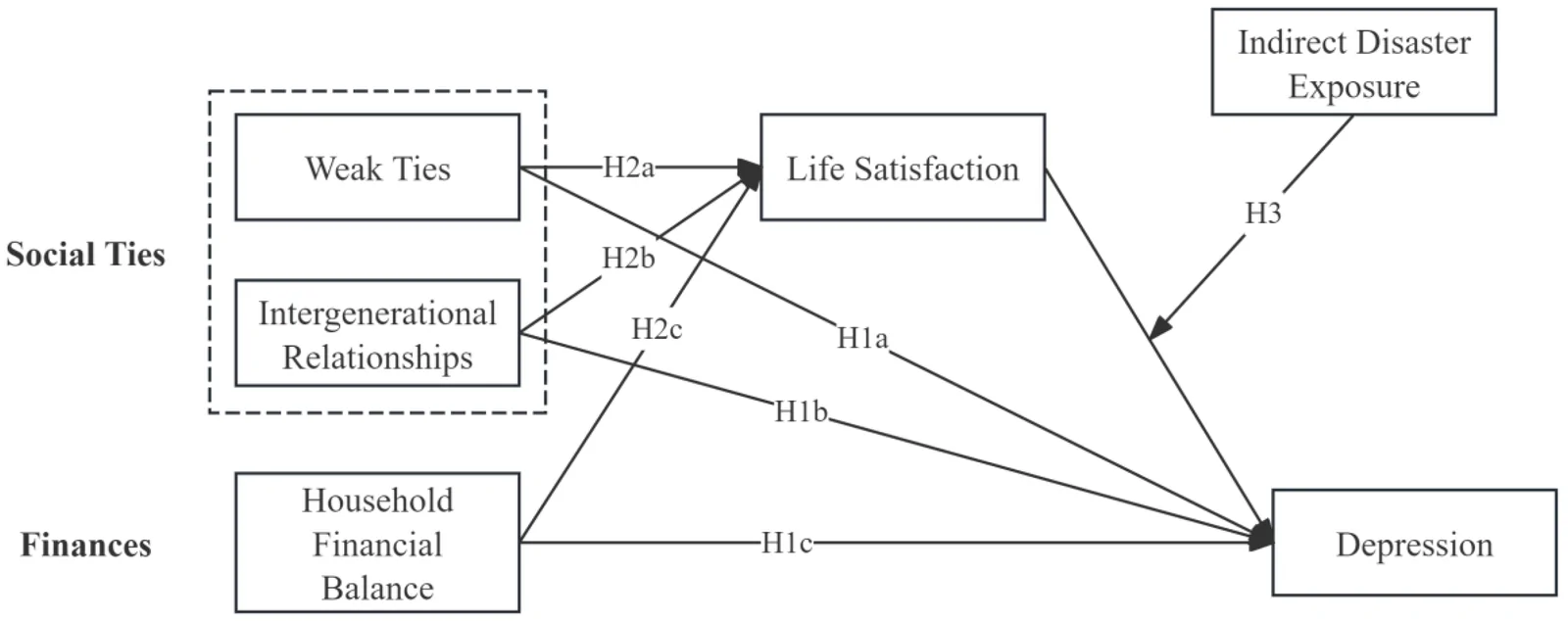
Theoretical framework.

In summary, this study proposes the theoretical framework shown in Figure 1.

## 3. Materials and Methods

### 3.1. Study Design and Participants

This study integrates open access data from multiple sources for comprehensive analysis. The datasets include the recent 2020 wave of CHARLS, the regional economic dataset from the National Bureau of Statistics of China, and the cumulative confirmed COVID-19 cases time-series data from the Chinese National Health Commission. Data processing and statistical analyses were performed using Stata 16.0 (StataCorp LLC, College Station, TX, USA).

CHARLS is a nationally conducted longitudinal survey designed primarily to collect health, socioeconomic, and family information from Chinese adults aged 45 years and older [21]. As part of its household-based design, the survey also interviews respondents’ spouses, including those younger than 45 years. This design allows household and intergenerational circumstances to be examined within a common survey framework. In this research, respondents and their spouses were retained to preserve the household structure of the dataset and to facilitate comparison with previous CHARLS-based studies that adopted the same analytic approach [5,21,22].

Notably, CHARLS 2020 includes unprecedented survey questions related to COVID-19, capturing subjective experiences and objective circumstances of middle-aged and older adults during this pandemic. These added questions provide critical insights into the impact of such a sudden disaster on the lives of middle-aged and older adults, enhancing the relevance of this study.

The analytic sample was constructed using a complete-case approach. Respondents were excluded if they had missing, invalid, or survey-defined non-substantive responses for the CES-D-10, weak ties, intergenerational relationships, household financial stability, life satisfaction, or any required covariate. In total, 3498 respondents had incomplete or invalid information on at least one required variable, resulting in a final analytic sample of 15,897 participants. No statistical imputation was performed. As a sensitivity analysis, the models were re-estimated among respondents aged 45 years or older.

### 3.2. Variables

#### 3.2.1. Dependent Variable

The dependent variable was measured using the 10-item Center for Epidemiologic Studies Depression Scale (CES-D-10) [23]. Scores ranged from 0 to 30, with higher scores indicating more severe depressive symptoms.

#### 3.2.2. Independent Variable

We operationalized weak and strong ties using contextually relevant proxy indicators available in the 2020 survey. These indicators were chosen because they indicate in the questions that the impact is affected by the COVID-19 pandemic, and this direct shock resulting from this unexpected event is particularly relevant to our research. It is worth noting that, due to the inability of the CHARLS questionnaire to accurately measure the degree of and changes in weak and strong relationships affected by emergencies, we chose the number of social activities and the changes in intergenerational relationships as proxy indicators for the two theoretical constructs.

Weak Ties: In line with prior research [5], weak ties were proxied by the number of social activities reported in the past month, including visiting friends, recreational activities, community activities, volunteering and educational activities. Higher scores indicate participation in a wider range of social activities and therefore more opportunities to maintain or develop low-intensity weak ties.

Intergenerational Relationships: This variable was measured using the CHARLS question “How has the COVID-19 outbreak this year changed your relationship with your children?” Respondents evaluated the perceived change in their relationship with each child. For respondents with multiple children, the average score was calculated. This item was used as a proxy for pandemic-related changes within a core strong tie domain. Higher scores indicate more positive perceived changes in intergenerational relationships.

Household financial stability: This variable was measured using the CHARLS question “Since the outbreak of the epidemic, do you think the income of your family is sufficient to cover their daily expenses?” Responses ranged from 1 (“very difficult”) to 4 (“very easy”), with higher scores indicating greater perceived ability of household income to cover daily expenses during the pandemic.

#### 3.2.3. Life Satisfaction

Life Satisfaction: The respondent’s subjective satisfaction with life, assessed using a five-category CHARLS item. For interpretability, we reverse-coded this item so that higher scores indicate greater life satisfaction. Responses were coded from −2 (“not at all satisfied”) to 2 (“completely satisfied”), with higher scores indicating greater life satisfaction.

#### 3.2.4. Moderator Variable

Indirect Disaster Exposure: The cumulative number of confirmed COVID-19 cases reported in each respondent’s city of residence in 2020 was used as a proxy for contextual indirect disaster exposure. City-level case counts were obtained from official reports issued by the Chinese National Health Commission and municipal health authorities. Considering that the CHARLS fieldwork lasted a relatively long period and interview timing varied across regions, cumulative case counts for the full calendar year were used. This measure may include cases reported after some participants completed their interviews. It should therefore be interpreted as overall city-level epidemic burden during 2020 rather than an exposure measure guaranteed to precede each assessment. Each respondent was assigned the case count for their city of residence. The city-level data were initially matched to individual CHARLS records by city name using Stata 16.0. Records that could not be matched automatically were manually reviewed and linked. This procedure successfully matched case-count data for the 125 cities represented in the analytic sample. To reduce right skewness and accommodate cities with no confirmed cases, the cumulative counts were transformed using log(cases + 1) before being included in the regression models. Standard errors were clustered at the city level to account for within-city correlation among respondents who shared the same contextual exposure value. Higher transformed values indicate a greater absolute city-level epidemic burden and were interpreted as greater indirect disaster exposure. This indicator reflects overall city-level epidemic burden rather than individual infection risk.

#### 3.2.5. Control Variables

Control variables included city-level GDP per capita, gender, age, education, marital status, and multimorbidity. City-level GDP per capita was measured for 2020 in Chinese yuan and transformed as log(GDP per capita + 1). Gender was coded as 1 for men and 2 for women. Age was entered as a continuous variable measured in years. In accordance with existing CHARLS research, educational level and marital status can be approximately regarded as continuous ordinal variables when used as covariates. Therefore, they were entered as single ordered covariates using the category codes reported in Table 1. Education was coded as 1 for less than lower secondary education, 2 for upper secondary education, 3 for vocational training, and 4 for tertiary education. Marital status was coded as 1 for married with the spouse present, 2 for married but temporarily not living with the spouse, 3 for separated, 4 for divorced, 5 for widowed, and 6 for never married. Multimorbidity was coded as 1 when a respondent reported two or more chronic conditions and 0 otherwise. The same set of control variables was included in all regression models.

### 3.3. Model Settings

Following the analytical framework developed by Wen and his colleagues [24,25], we constructed the models presented in Equations (1)–(4). Among them, Equations (1) and (2) are designed to examine the associations of weak ties, intergenerational relationships, and household financial stability with depression levels and life satisfaction, respectively. Equations (1)–(3) were considered jointly to describe the proposed cross-sectional indirect associations involving life satisfaction. Formal statistical inference was based on the estimated indirect associations and their bootstrap confidence intervals.

(1) $${\;G}_{i}=a_{0}+a_{1}C_{i}+a_{2}D_{i}+a_{3}E_{i}+a_{4}F_{i}+a_{5}H_{i}+\varepsilon_{i}$$

(2) $${\;B}_{i}=b_{0}+b_{1}C_{i}+b_{2}D_{i}+b_{3}E_{i}+b_{4}F_{i}+b_{5}H_{i}+\varepsilon_{i}$$

(3) $${\;G}_{i}=c_{0}+c_{1}B_{i}+c_{2}C_{i}+c_{3}D_{i}+c_{4}E_{i}+c_{5}F_{i}+c_{6}H_{i}+\varepsilon_{i}$$

In Equations (1)–(3), *G*_i_ represents the depression level, *B*_i_ represents life satisfaction, *C*_i_ represents weak ties, *D*_i_ represents intergenerational relationships, *E*_i_ represents household financial stability, *F*_i_ represents cumulative confirmed cases, and *H*_i_ and *ε*_i_ represent control variables and error terms, respectively. For formal statistical inference, the indirect associations were estimated using 5000 bootstrap resamples. In accordance with regression settings, two-sided 90% confidence intervals were calculated from the bootstrap standard errors. An indirect association was considered statistically significant when its 90% confidence interval did not include 0.

(4) $${\;G}_{i}=d_{0}+d_{1}B_{i}+d_{2}C_{i}+d_{3}D_{i}+d_{4}E_{i}+d_{5}B_{i}\times F_{i}+d_{6}F_{i}{+d}_{7}H_{i}+\varepsilon_{i}$$

Equation (4) tests whether the association between life satisfaction and depressive symptoms varies with cumulative confirmed cases. A significant *d*_5_ indicates a statistically significant interaction between life satisfaction and indirect disaster exposure.

Given the cross-sectional design, the estimates above should be interpreted as statistical decompositions of concurrent associations rather than evidence of temporal or causal mediation. Also, CHARLS employs a multistage, stratified probability sampling design. The present analyses did not apply survey weights, so the regression estimates should not be interpreted as nationally representative population estimates.

The CES-D-10 total score was treated as a continuous measure of depressive symptoms, ranging from 0 to 30 and thus containing 31 possible values. Therefore, OLS was selected because it allows the full variation in depressive symptom scores to be retained and the coefficients to be interpreted as mean score differences. The linear specification also allowed the indirect associations and interaction effects to be estimated within a consistent analytical framework. Considering that COVID-19 exposure and GDP per capita were measured at the city level, participants living in the same city may share the same contextual values. Therefore, all regression models used standard errors clustered at the city level to account for within-city dependence across the 125 cities. Bootstrap confidence intervals for the estimated indirect associations were also obtained by resampling at the city-cluster level.

## 4. Results

### 4.1. Descriptive Statistics

According to the descriptive statistics for the variables presented in Table 1, the mean of the CES-D score is relatively low, but the standard deviation is large. This indicates that while the overall level of depression among residents is low, there is significant variation in individual depression levels. A majority of respondents (89.5%) claimed to be at least somewhat satisfied with their current life. Over half of the households (58.2%) had experienced varying degrees of difficulty in managing daily expenses since the onset of the emergency. Furthermore, the variable representing the impact of the emergency on intergenerational relationships has a mean of 0.005 and a standard deviation of 0.244, suggesting that the emergency had relatively little effect on intergenerational relationships.

### 4.2. Hypothesis Testing

The regression results of this study are presented in Table 2. Model 1 examines the impact of household financial stability, social activity participation, and changes in intergenerational relationships on depression among middle-aged and older adults during an emergency. Models 2 and 3 introduce life satisfaction to estimate the proposed cross-sectional indirect associations. Model 4 further incorporates an interaction term between indirect disaster exposure and life satisfaction to examine whether the association between life satisfaction and depressive symptoms varies with indirect disaster exposure.

#### 4.2.1. Main Effects

As shown in Table 2, Model 1, the results indicate that social activity participation (b = −0.277, *p* < 0.01) and changes in intergenerational relationships (b = −1.026, *p* < 0.01) are negatively associated with depression among middle-aged and older adults during an emergency. This suggests that improved weak ties and enhanced intergenerational relationships are significantly associated with lower depression levels among middle-aged and older adults, thus supporting hypotheses H1a and H1b. The regression analysis (Table 2, Model 1) also revealed that household financial stability (b = −2.062, *p* < 0.01) has a significant negative association with depression among middle-aged and older adults. This suggests that improved household financial stability was significantly associated with lower depression levels among middle-aged and older adults, consistent with H1c.

#### 4.2.2. Results of Cross-Sectional Indirect Associations Involving Life Satisfaction

The results show that household financial stability, social activity participation, and changes in intergenerational relationships are significantly positively associated with life satisfaction. As shown in Table 2, Model 3, life satisfaction (b = −2.594, *p* < 0.01) has a significant negative association with depression among middle-aged and older adults. Compared with Model 1, the absolute coefficients of the three focal predictors were smaller after life satisfaction was included in Model 3, while R^2^ increased from 0.1687 to 0.2635. These results motivated the formal examination of the indirect associations reported in Table 3.

As shown in Table 3, Bootstrap tests with 5000 resamples indicate the statistical indirect contribution of life satisfaction. The 90% confidence intervals for the cross-sectional indirect association of household financial stability, social activity participation, and changes in intergenerational relationships did not include zero, supporting H2a, H2b, and H2c.

#### 4.2.3. Interaction Between Life Satisfaction and Indirect Disaster Exposure

As shown in Model 4 of Table 2, the interaction term between life satisfaction and indirect disaster exposure was negative and significant (b = −0.134, *p* < 0.01), indicating that the negative association between life satisfaction and depressive symptoms varied according to the level of indirect disaster exposure. However, R^2^ increased only from 0.2635 to 0.2641 after the interaction was added (ΔR^2^ = 0.0006), indicating a small incremental contribution despite statistical significance.

**Figure 2:**
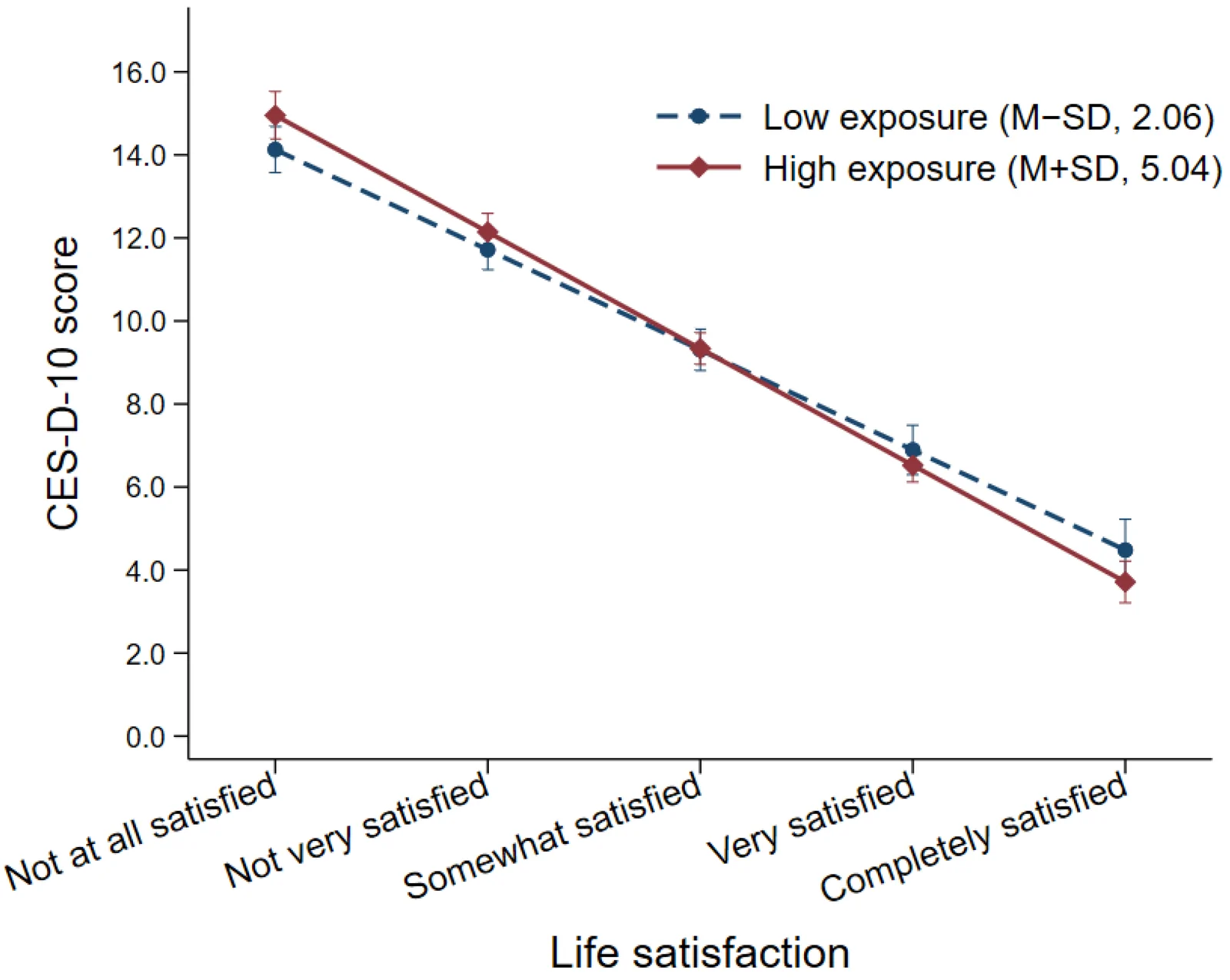
The moderating effect of indirect disaster exposure between life satisfaction and depression.

As illustrated in Figure 2, the slope of the “High Exposure” (one standard deviation above the mean, 5.04) line is steeper (b = −2.811, SE = 0.096, 95% CI [−3.001, −2.621], *p* < 0.01) than that of the “Low Exposure” (one standard deviation below the mean, 2.06) line (b = −2.412, SE = 0.108, 95% CI [−2.625, −2.199], *p* < 0.01). The predictions and their 95% confidence intervals are also presented in Figure 2. This shows that the negative relationship between life satisfaction and depression becomes stronger under high exposure. This difference in slopes is consistent with the significant interaction term and supports H3.

Simultaneously, household financial stability, social activity participation, and intergenerational relationships remain negatively associated with depression at the 5% level, further confirming hypotheses H1a, H1b, and H1c.

### 4.3. Robustness Tests

To further confirm the reliability and generalizability of our research results, we conducted two robustness checks, including imposing a stricter age restriction and an alternative estimation method.

First, the baseline regression retained data from both respondents and their spouses, which included a small portion of samples under the age of 45 to facilitate comparison with the previous CHARLS research results. Here, we excluded the samples under the age of 45 to more clearly explore the applicability of our research conclusions to the middle-aged and older population. The regression results are shown in Table 4, Model 1. The direction and significance of all variables remained unchanged, indicating the robustness of our model.

Second, we replaced the original OLS model with a binary logistic model. Specifically, CES-D-10 scores of 0–9 were coded as below the screening threshold, while scores of 10–30 were coded as indicating relevant depressive symptoms [23,26]. The result from logistic regression, shown in Table 4, Model 2, indicates that social activity participation, household financial stability, life satisfaction, and the interaction term retained their expected directions and were statistically significant. Perceived intergenerational relationship change was significant at the 10% level, while the main effect of indirect disaster exposure was not statistically significant. Thus, the results from both robustness checks are consistent with the previous findings, confirming the model’s robustness.

## 5. Discussion

This study employs a cross-sectional regression framework incorporating indirect associations and an interaction term to investigate the psychosocial patterns of depression among middle-aged and older adults in China during an emergency. Our findings not only examine the roles of social ties and finances but also provide a novel perspective by showing that the association between life satisfaction and depressive symptoms varied slightly with indirect disaster exposure. This insight underscores the importance of integrating individual resources with broader contextual stressors to fully understand mental health during an emergency.

### 5.1. The Roles of Social Ties and Finances

Our baseline results align with and extend Social Support Theory and COR theory, delineating distinct yet complementary pathways through which social ties and financial resources are associated with depression among middle-aged and older adults during an emergency.

Broader social activity participation was associated with fewer depressive symptoms. During lockdowns and social distancing, such activities may have provided opportunities to remain connected beyond close family networks [27]. Our findings are consistent with prior research linking broader and more diverse social networks to fewer depressive symptoms [16,28]. The observed associations between network scale and depression symptoms underscore the importance of social activity participation during public health emergencies.

More positive perceived changes in intergenerational relationships, used as a proxy for strong ties, were associated with fewer depressive symptoms, particularly within the Chinese cultural context. Our finding that improvements in these relationships during an emergency are associated with lower depression deepens the existing understanding of strong ties [17]. In a society deeply influenced by Confucian filial piety and the tradition of “raising children for old age” [14], enhanced intergenerational bonds likely provided not only emotional solace but also a reinforced sense of familial duty and security. This aligns with studies showing that positive intergenerational relationships are a key source of family social support and are negatively associated with depressive symptoms in middle-aged and older adults [4].

Consistent with COR theory’s loss principle [18,19], household financial stability emerged as a critical material resource, as the emergency context amplified the threat of resource loss. The fear and actuality of financial depletion constitute a direct manifestation of COR’s “loss spirals.” Household financial stability is of extreme importance in older adults coming from different cultural contexts. Evidence from England links non-mortgage household debt with poorer mental well-being among older adults, whereas Australian research indicates that financial well-being varies with consumption patterns, financial literacy, health, housing conditions, and socioeconomic resources [29,30]. Longitudinal evidence from China further corroborates this relationship, demonstrating that household financial debt significantly exacerbates depressive symptoms [31]. Thus, our findings suggest that financial stability acts not merely as an economic indicator but as a psychological resource. It is negatively associated with anxiety and helplessness which fuel depression, a relationship consistently observed between economic hardship and mental health issues [32]. This pattern is particularly salient during crises, as evidenced by the COVID-19 emergency, in which household financial difficulties were linked to severe emotional distress and heightened suicide risk among vulnerable groups, underscoring how financial strain erodes emotional security [33]. By securing material resources, middle-aged and older adults can conserve psychological resources otherwise expended on financial worries, thus safeguarding their mental well-being amidst a crisis.

### 5.2. The Role of Life Satisfaction and Indirect Disaster Exposure

The study identified significant indirect associations involving life satisfaction in the relationships between social and financial resources and depressive symptoms among middle-aged and older adults. This pattern is consistent with the buffering hypothesis [34], whereby both relational support and material security are associated with more favorable evaluations of life [8]. The elevated life satisfaction was, in turn, associated with fewer depressive symptoms [10]. This result empirically substantiates prior findings that informal social support and material security are closely linked to life satisfaction [35,36]. More importantly, life satisfaction statistically contributed to the associations between external shocks and depressive symptoms, akin to findings by Yang et al., through which external resources confer mental health benefits and adversities translate into psychological distress [20]. These statistical indirect associations were consistently observed across different resource types.

Furthermore, the association between life satisfaction and depression symptoms varied significantly across different levels of indirect disaster exposure. Specifically, the negative association was somewhat stronger at higher exposure levels, although the interaction added very little explanatory power (ΔR^2^ = 0.0006). This counter-intuitive pattern is consistent with the possibility that under the threat of exposure, life satisfaction transforms from a stable trait into a psychological resource. For individuals with high life satisfaction, this resource becomes mobilized against the pervasive threat, enhancing the negative association. Conversely, for those with low life satisfaction, greater external threat may place additional strain on their limited psychological resources, corresponding to higher depressive symptom levels. This nuanced interaction between individual psychological resources and environmental context provides important insights into how mental health outcomes are jointly determined during emergencies.

### 5.3. Practical Implications

Taken together, the findings point to an integrated approach to protecting middle-aged and older adults’ mental health during an emergency. First, make financial help easy to use, such as by providing short-term cash or utility support and reliable delivery of essentials with senior-friendly access. Second, keep everyday contact going. Offer brief digital help, helplines, and loaner devices or data so middle-aged and older adults can reach family, health providers, and community groups. Third, when public health conditions permit, low-risk activities should be resumed in outdoor or well-ventilated spaces, to alleviate anxiety and restore a sense of normalcy. Finally, given that higher emergency severity strengthens the association between life satisfaction and depression, these measures deserve particular attention in harder-hit cities, although the incremental explanatory power of this interaction is modest.

### 5.4. Limitations and Prospects

This study has several limitations that also point to directions for future work. First, our model centers on social ties, household financial stability, and emergency characteristics. Depression during a crisis is multifaceted, and unobserved factors may matter. Future research should broaden the set of determinants and pathways examined. Second, following recent methodological reflections on mental-health assessment, reliance on self-reported rating scales may entail biases. Pre-existing depressive symptoms and even emotional state may affect self-reporting results [37,38]. Third, to fully account for the impact of emergencies, the number of social activities and changes in intergenerational relationships were used as proxy measures for weak ties and strong ties, respectively, which may introduce biases. Further research should utilize more direct variable constructions in emergency situations to measure the impact of relationship strength. Although the CES-D-10 is widely used, its results may differ from clinical diagnoses; future work should incorporate more objective assessments or link to more rigorous data sources to improve measurement accuracy. Fourth, to examine emergencies’ effects with large-scale survey data, we use the fifth wave of CHARLS. Considering that the analysis was cross-sectional, temporal ordering and causal effect could not be established. As new waves and complementary datasets become available, future research should integrate these sources for further analysis. Fifth, due to the long duration of the CHARLS interviews, the use of full-year 2020 cumulative case counts may introduce temporal mismatch because the measure could include cases reported after some respondents completed their interviews. Future studies should align city-level exposure windows with individual interview dates.

## 6. Conclusions

This study demonstrates that broader social activity participation, more positive perceived changes in intergenerational relationships, and better household finances were associated with fewer depressive symptoms among middle-aged and older adults during public health emergencies. Life satisfaction statistically contributed to these relationships. Furthermore, the negative association between life satisfaction and depression was slightly stronger at higher levels of indirect disaster exposure. These findings identify an integrated pattern of individual and city-level associations, underscoring how macro-level stressors are linked to psychological vulnerabilities.

## Figures and Tables

**Table 1:** Descriptive statistics for each variable.

Variable	
CES-D, Mean ± Standard deviation	8.606 ± 6.441
Life satisfaction, *n* (%)	
Not at all satisfied	401, (2.5%)
Not very satisfied	1279, (8.0%)
Somewhat satisfied	8553, (53.8%)
Very satisfied	4894, (30.8%)
Completely satisfied	770, (4.9%)
Household financial stability, *n* (%)	
Very difficult	2121, (13.3%)
Somewhat difficult	7133, (44.9%)
Easy	5992, (37.7%)
Very Easy	651, (4.1%)
Weak ties, Mean ± Standard deviation	0.829 ± 1.047
Intergenerational relationships, Mean ± Standard deviation	0.005 ± 0.244
GDP_PC, Mean ± Standard deviation	10.901 ± 0.445
Indirect disaster exposure, Mean ± Standard deviation	3.548 ± 1.489
Gender, *n* (%)	
Male	7543, (47.4%)
Female	8354, (52.6%)
Age, Mean ± Standard deviation	60.527 ± 9.332
<45	205, (1.3%)
45–60	7450, (46.9%)
61–75	6979, (43.9%)
>75	1263, (7.9%)
Education, *n* (%)	
Less than lower secondary education	13,721, (86.3%)
Upper secondary	1426, (9.0%)
Vocational training	620, (3.9%)
Tertiary education	130, (0.8%)
Marriage, *n* (%)	
Married with spouse present	12,449, (78.3%)
Married but not living with spouse temporarily	1327, (8.3%)
Separated	58, (0.4%)
Divorced	210, (1.3%)
Widowed	1840, (11.6%)
Never married	13, (<0.1%)
Multimorbidity, *n* (%)	
No multimorbidity	13,897, (87.4%)
With multimorbidity	2000, (12.6%)

**Table 2:** Regression results.

Variables	Model 1	Model 2	Model 3	Model 4
	CES-D	Satisfaction	CES-D	CES-D
Satisfaction			−2.594 ***	−2.136 ***
			(0.077)	(0.192)
Finance	−2.062 ***	0.163 ***	−1.640 ***	−1.637 ***
	(0.082)	(0.010)	(0.084)	(0.084)
Weak ties	−0.277 ***	0.0134 *	−0.242 ***	−0.242 ***
	(0.051)	(0.007)	(0.047)	(0.047)
Intergen	−1.026 ***	0.184 ***	−0.550 ***	−0.536 **
	(0.220)	(0.026)	(0.206)	(0.207)
Exposure	0.006	−0.013	−0.027	0.010
	(0.134)	(0.008)	(0.131)	(0.127)
GDP_PC	−1.270 ***	0.009	−1.245 ***	−1.253 ***
	(0.340)	(0.031)	(0.313)	(0.313)
Gender	2.191 ***	−0.023 *	2.131 ***	2.130 ***
	(0.118)	(0.013)	(0.102)	(0.101)
Age	0.073 ***	0.003 ***	0.082 ***	0.082 ***
	(0.007)	(0.001)	(0.006)	(0.006)
Education	−0.835 ***	−0.046 ***	−0.953 ***	−0.954 ***
	(0.092)	(0.013)	(0.087)	(0.087)
Marriage	0.316 ***	−0.021 ***	0.263 ***	0.263 ***
	(0.041)	(0.006)	(0.040)	(0.040)
Multimorbidity	2.571 ***	−0.154 ***	2.171 ***	2.177 ***
	(0.154)	(0.020)	(0.133)	(0.134)
Satisfaction × Exposure				−0.134 ***
				(0.048)
Constant	19.044 ***	−0.282	18.312 ***	18.234 ***
	(3.454)	(0.335)	(3.134)	(3.137)
Observations	15,897	15,897	15,897	15,897
R^2^	0.1687	0.0387	0.2635	0.2641

Note: Standard errors in parentheses, * *p* < 0.1, ** *p* < 0.05, *** *p* < 0.01. Standard errors were clustered at the city level and are reported in parentheses.

**Table 3:** Bootstrap testing results.

		Coefficient	Std.err	*z*	*p* > |*z*|	[90% CI]	
	
Weak ties	Indirect association	−0.035	0.018	−1.90	0.057	−0.065	−0.005
	Direct association	−0.242	0.048	−5.04	0.000	−0.322	−0.163
Intergen	Indirect association	−0.476	0.069	−6.89	0.000	−0.590	−0.363
	Direct association	−0.550	0.207	−2.66	0.008	−0.890	−0.210
Finance	Indirect association	−0.422	0.030	−13.92	0.000	−0.471	−0.372
	Direct association	−1.640	0.083	−19.74	0.000	−1.777	−1.504

**Table 4:** Results of the Robustness tests.

Variables	Age ≥ 45	Logit
	Model 1	Model 2
	CES-D	Depression
Satisfaction	−2.138 ***	−0.598 ***
	(0.190)	(0.094)
Finance	−1.644 ***	−0.512 ***
	(0.084)	(0.031)
Weak ties	−0.241 ***	−0.054 ***
	(0.048)	(0.019)
Intergen	−0.513 **	−0.160 *
	(0.208)	(0.086)
GDP_PC	−1.261 ***	−0.462 ***
	(0.315)	(0.111)
Exposure	0.013	0.013
	(0.126)	(0.042)
Satisfaction × Exposure	−0.132 ***	−0.055 **
	(0.047)	(0.024)
Control Variables	Yes	Yes
Observations	15,692	15,897
R^2^/McFadden’s pseudo-R^2^	0.2642	0.1421

Note: Standard errors in parentheses, * *p* < 0.1, ** *p* < 0.05, *** *p* < 0.01. Standard errors were clustered at the city level and are reported in parentheses.

## Data Availability

The CHARLS data used in this study are available upon application through the official China Health and Retirement Longitudinal Study website.

## References

[1] GBD 2023 Mental Disorder Collaborators (2026). Updated trends in the global prevalence and burden of mental disorders, 1990–2023: A systematic analysis for the Global Burden of Disease Study 2023. Lancet.

[2] Rogers J.P., Chesney E., Oliver D., Pollak T.A., McGuire P., Fusar-Poli P., Zandi M.S., Lewis G., David A.S. (2020). Psychiatric and neuropsychiatric presentations associated with severe coronavirus infections: A systematic review and meta-analysis with comparison to the COVID-19 pandemic. Lancet Psychiatry.

[3] Parker G., Lie D., Siskind D.J., Martin-Khan M., Raphael B., Crompton D., Kisely S. (2016). Mental health implications for older adults after natural disasters: A systematic review and meta-analysis. Int. Psychogeriatr..

[4] Yang M., Wang H., Yao J. (2022). Relationship between intergenerational emotional support and subjective well-being among elderly migrants in China: The mediating role of loneliness and self-esteem. Int. J. Environ. Res. Public Health.

[5] Wang J., Zhang J., Lin H., Han Y., Tu J., Nie X. (2023). Economic development, weak ties, and depression: Evidence from China. J. Affect. Disord..

[6] Tragantzopoulou P., Giannouli V. (2021). Social isolation and loneliness in old age: Exploring their role in mental and physical health. Psychiatriki.

[7] Ettman C.K., Abdalla S.M., Cohen G.H., Sampson L., Vivier P.M., Galea S. (2021). Low assets and financial stressors associated with higher depression during COVID-19 in a nationally representative sample of US adults. J. Epidemiol. Community Health.

[8] Diener E., Lucas R.E., Oishi S. (2018). Advances and open questions in the science of subjective well-being. Collabra Psychol..

[9] Tan J.J.X., Kraus M.W., Carpenter N.C., Adler N.E. (2020). The association between objective and subjective socioeconomic status and subjective well-being: A meta-analytic review. Psychol. Bull..

[10] Kim E.S., Delaney S.W., Tay L., Chen Y., Diener E., VanderWeele T.J. (2021). Life satisfaction and subsequent physical, behavioral, and psychosocial health in older adults. Milbank Q..

[11] Merdjanoff A.A., Abramson D.M., Piltch-Loeb R., Findley P., Peek L., Beedasy J., Park Y.S., Sury J., Meltzer G.Y. (2022). Examining the dose–response relationship: Applying the Disaster Exposure Matrix to understand the mental health impacts of Hurricane Sandy. Clin. Soc. Work J..

[12] Guo Y., Sims O.T., Qin W., Yang F. (2021). Factors associated with symptoms of depression and psychological distress during the COVID-19 pandemic. Behav. Sci..

[13] National Bureau of Statistics of China Statistical Communique of the People’s Republic of China on the 2023 National Economic and Social Development. https://www.stats.gov.cn/english/PressRelease/202402/t20240228_1947918.html.

[14] Guo Q., Gao X., Sun F., Feng N. (2020). Filial piety and intergenerational ambivalence among mother–adult child dyads in rural China. Ageing Soc..

[15] Wang Y., Zhou D., Wang C. (2024). Influences of public health emergency and social isolation on older adults’ wellbeing: Evidence from a longitudinal study. Front. Public Health.

[16] Kim H.H. (2017). “Strength of weak ties,” neighborhood ethnic heterogeneity, and depressive symptoms among adults: A multilevel analysis of Korean General Social Survey (KGSS) 2012. Soc. Sci..

[17] Leach L.S., Butterworth P., Olesen S.C., Mackinnon A. (2013). Relationship quality and levels of depression and anxiety in a large population-based survey. Soc. Psychiatry Psychiatr. Epidemiol..

[18] Hobfoll S.E. (1989). Conservation of resources: A new attempt at conceptualizing stress. Am. Psychol..

[19] Hobfoll S.E. (2001). The influence of culture, community, and the nested-self in the stress process: Advancing conservation of resources theory. Appl. Psychol..

[20] Yang Y., Liu Y., Jiang Z., Mo J., Wang C., Yang Y., Jia X., Lin L. (2021). Negative affect and life satisfaction mediate the association between negative life events and suicidal ideation in college students. Psychol. Health Med..

[21] Zhao Y., Hu Y., Smith J.P., Strauss J., Yang G. (2014). Cohort profile: The China Health and Retirement Longitudinal Study (CHARLS). Int. J. Epidemiol..

[22] Liu J., Liu Z., Ding P. (2026). Exploring depression in older adults amid crises: A moderated mediation analysis through the lens of conservation of resources theory. Int. J. Soc. Psychiatry.

[23] Andresen E.M., Malmgren J.A., Carter W.B., Patrick D.L. (1994). Screening for depression in well older adults: Evaluation of a short form of the CES-D. Am. J. Prev. Med..

[24] Wen Z., Ye B. (2014). Analyses of mediating effects: The development of methods and models. Adv. Psychol. Sci..

[25] Wen Z., Zhang L., Hou J. (2006). Mediated moderator and moderated mediator. Acta Psychol. Sin..

[26] Fu H., Si L., Guo R. (2022). What is the optimal cut-off point of the 10-item Center for Epidemiologic Studies Depression Scale for screening depression among Chinese individuals aged 45 and over? An exploration using latent profile analysis. Front. Psychiatry.

[27] Granovetter M.S. (1973). The strength of weak ties. Am. J. Sociol..

[28] Reiner A., Steinhoff P. (2024). The association of social networks and depression in community-dwelling older adults: A systematic review. Syst. Rev..

[29] Hiilamo A. (2020). Debt matters? Mental wellbeing of older adults with household debt in England. SSM Popul. Health.

[30] Xue R., Gepp A., O’Neill T.J., Stern S., Vanstone B.J. (2020). Financial well-being amongst elderly Australians: The role of consumption patterns and financial literacy. Account. Financ..

[31] Hu M., Nie W., Song J., Wang T., Ye X. (2023). Relationship between household financial debt and depressive symptoms: A longitudinal study in China. BMJ Open.

[32] Kim D. (2021). Financial hardship and social assistance as determinants of mental health and food and housing insecurity during the COVID-19 pandemic in the United States. SSM Popul. Health.

[33] Park J.Y., Ha J. (2023). Impact of emotional state and suicidal intentions on suicide attempts among Korean adolescents with household financial difficulties following the outbreak of COVID-19: A cross-sectional study. Medicine.

[34] Cohen S., Wills T.A. (1985). Stress, social support, and the buffering hypothesis. Psychol. Bull..

[35] Dong Y., Cheng L., Cao H. (2024). Impact of informal social support on the mental health of older adults. Front. Public Health.

[36] Bridson L., Robinson E., Putra I.G.N.E. (2024). Financial-related discrimination and socioeconomic inequalities in psychological well-being related measures: A longitudinal study. BMC Public Health.

[37] Giannouli V., Tsolaki M. (2022). Is negative affect associated with deficits in financial capacity in nondepressed older adults? A preliminary study. J. Affect. Disord. Rep..

[38] Giannouli V., Stamovlasis D., Tsolaki M. (2022). Longitudinal study of depression on amnestic mild cognitive impairment and financial capacity. Clin. Gerontol..

